# Optimization of Ornithopter Energy Efficiency Through Spring-Induced Harmonic Motion

**DOI:** 10.3390/biomimetics11030207

**Published:** 2026-03-13

**Authors:** Jimin Kim, Ji-Chul Ryu

**Affiliations:** 1 North London Collegiate School Jeju, Seogwipo-si 63644, Republic of Korea; jmkim27@pupils.nlcsjeju.kr; 2 Department of Mechanical Engineering, Northern Illinois University, Dekalb, IL 60115, USA

**Keywords:** ornithopter, flapping-wing UAV, resonance tuning, harmonic oscillator, energy efficiency

## Abstract

Ornithopters generate lift and thrust through periodic flapping-wing motion. While control-based optimization has been widely studied to improve the flight efficiency of ornithopters, passive mechanical tuning remains underexplored. This study investigates whether integrating a lightweight torsional spring can passively tune a flapping-wing system toward resonance to reduce input power and enhance aerodynamic performance. We evaluated springs of different stiffness on a 3D-printed, motor-driven flapping rig, recording input voltage and current as well as flapping frequency and thrust. Wing kinematics were analyzed using high-speed video, and free-oscillation tests identified a resonant period of ~0.14 s (~7.1 Hz). Experimental results show that an optimally tuned spring-assisted system achieves up to a threefold improvement in thrust efficiency and up to a twofold improvement in kinematic efficiency, compared to the no-spring baseline. Indoor flight tests using a commercial ornithopter (MetaFly) confirmed the improvement, showing a 12.8% increase in average endurance. The spring-assisted configuration also produced smoother stroke reversals, consistent with reduced energy losses. These results demonstrate that a low-complexity, lightweight torsional spring tuned near resonance can provide an effective passive means to enhance both energy efficiency and aerodynamic output in flapping-wing UAVs, serving as a practical, low-cost complement to control-based optimization methods.

## 1. Introduction

Ornithopters are unmanned aerial vehicles that generate thrust and lift via bird-like flapping. These aerial vehicles have attracted significant interest for applications in constrained environments such as search-and-rescue, surveillance, and environmental monitoring. However, typical battery-powered ornithopters achieve only 5–15 min of flight per charge due to the high power required for continuous actuation of the wings [1,2,3,4]. Improving energy efficiency without increasing design and control complexity remains challenging for practical deployment.

Birds store elastic energy in tendons and release it during each stroke cycle. For example, in pigeons, elastic recoil in the supracoracoideus muscle (SUPRA) can contribute up to about 60% of net mechanical work during upstroke, enhancing efficiency [5]. Recent overviews of avian flight biomechanics synthesize how tendon-mediated elastic storage and recoil reduce mechanical work during stroke reversals [6]. Inspired by this biomechanics, resonant flapping has been recognized as a path to improve efficiency in small flapping-wing UAVs; when the driving frequency is tuned near the natural frequency of the wing linkage, the required input power is reduced [7]. Design and scaling analyses further define the relationship among stiffness, inertia, and frequency for efficient resonant flapping and outline the system-level energetics of resonance-based actuation [8].

In engineering applications, elastic elements such as springs have been integrated into flapping-wing mechanisms to reduce inertial power and exploit resonance. Several studies on flapping-wing micro-air vehicles (FWMAVs) have demonstrated that appropriately tuned elastic components can lower electrical input power or lift output by driving the system near its mechanical resonance [9]. More recent work has developed spring–wing models that relate wing inertia, stiffness, and internal damping to resonant behavior and power consumption, providing a general framework for when elastic storage is beneficial [10]. Building on these foundations, several bioinspired prototypes with compliant or spring-assisted drives have reported smoother stroke reversals and improved energetic performance in hover or low-speed flight [11,12].

In addition to using discrete coil springs in the transmission, several FWMAVs have integrated elastic elements directly into the wing joints or pitch hinges, so that the joint both transmits motion and stores elastic energy. Flexure-based or torsional elastic hinges have been shown to enable passive wing pitching and resonance while reducing the added mass and integration complexity associated with conventional coil springs [13,14].

While these studies demonstrate the benefits of passive mechanisms, prior work has predominantly emphasized active efficiency strategies such as computational trajectory optimization—planning gliding/flapping sequences or shaping motion under nonlinear dynamics [15,16,17]. Examples include transition-trajectory optimization for FWMAVs and learning-based planners that accelerate energy-efficient path generation [18,19]. While many experimental studies report improvements in terms of smoother reversals or lift generation [20,21,22,23,24], few quantify energy efficiency across different stiffness configurations or validate experimental results in actual flight.

To address this gap, this study integrates analytical modeling, controlled bench-top experiments, and flight validation. Motivated by avian biomechanics, we evaluated whether adding a lightweight torsional spring to a flapping mechanism can passively tune the wing system toward resonance and measurably reduce power consumption. The wing mechanism was modeled as a harmonic oscillator to provide a framework for interpreting resonance effects. Therefore, the primary contributions of this work are as follows: (i) systematic characterization of stiffness-dependent performance on a flapping rig by varying the torsional-spring stiffness while measuring electrical input power, flapping frequency, and generated thrust; and (ii) translation of these quantitative bench-scale measurements to endurance validation on a commercial ornithopter platform, demonstrating system-level energy-efficiency gains. By isolating the spring’s contribution to energy use and aerodynamic performance, this work demonstrates a low-complexity, passive method to improve energy efficiency in flapping-wing UAVs and provides practical guidance for selecting stiffness in future designs.

## 2. Background

### 2.1. Wing Mechanism of Ornithopter

Ornithopter wings replicate the flapping motion of birds using lightweight materials with a flexible frame and aerodynamic surface to generate lift and thrust while minimizing inertia. In a typical wing mechanism, a rotary motor drives a crank-connecting rod linkage that converts rotation into periodic up-and-down wing motion, mimicking natural bird flight.

**Figure 1:**
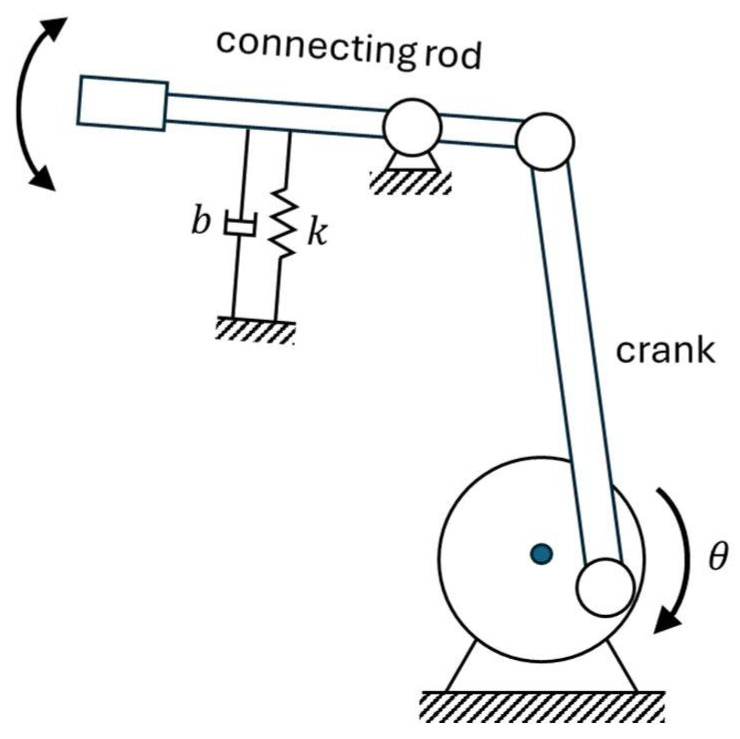
Schematic of the Ornithopter Joint Mechanism.

In our proposed configuration, a spring mechanism is integrated into the flapping linkage to store elastic energy during the downward stroke and release it during the reversal. Without the spring, the motor must supply all the torque to move the wings, especially to overcome the resistance needed to reverse the wing’s direction during each stroke, leading to higher energy consumption. Therefore, incorporating a spring helps the stroke reversal, reducing the load on the motor and improving overall energy efficiency. Furthermore, operating near resonance significantly reduces energy loss, enhancing flight efficiency and endurance. The schematic of the ornithopter flapping linkage mechanism integrated with a torsional spring is illustrated in Figure 1.

### 2.2. Mathematical Modeling of Forced Harmonic Oscillations

To understand how the addition of a torsional spring can improve flapping energy efficiency, the wing linkage can be modeled as a forced, damped harmonic oscillator. The rotational dynamics is governed by

(1) $$I\ddot{\theta }+b\dot{\theta }+k\theta =T_{0}cos(\omega t)$$

where *I* denotes the moment of inertia of the rotating wing system, *b* the damping coefficient, *k* the torsional spring constant, $T_{0}$ the amplitude of the driving torque, and ω the driving frequency. Defining the natural frequency $\omega_{n}=\sqrt{k/I}$ and the damping coefficient per unit inertia β=b/I, Equation (1) can be rewritten as

(2) $$\ddot{\theta }+\beta \;\dot{\theta }+\omega_{n}^{2}\theta =\frac{T_{0}}{I}cos(\omega t)$$

By solving the equation, the steady-state amplitude A of the system’s oscillatory response under the sinusoidal driving force is given by

(3) $$A=\frac{T_{0}/I}{\sqrt{{\left(\omega_{n}^{2}-\omega^{2}\right)}^{2}+{\left(\beta \omega \right)}^{2}}}$$

Maximum amplitude occurs when the driving frequency ω approaches the natural frequency $\omega_{n}$, corresponding to resonance.

To analyze the effect of the addition of a torsional spring, we first assume k=0, corresponding to the absence of the spring. Under this condition, the system operates purely as a damped harmonic oscillator driven by an external torque. By comparing the amplitudes of the system with k=0 and k>0, we can determine the conditions under which the inclusion of the spring enhances the amplitude.

Without a torsional spring, which is k=0, i.e., $\omega_{n}=0$, the amplitude in Equation (3) reduces to

(4) $$A_{0}=\frac{T_{0}/I}{\sqrt{\omega^{4}+(\beta \omega {)}^{2}}}$$

To ensure the spring yields a larger oscillation amplitude than the no-spring case, we need to satisfy $A>A_{0}$, leading to the inequality

(5) $$\frac{1}{(\omega_{n}^{2}-\omega^{2}{)}^{2}+(\beta \omega {)}^{2}}>\frac{1}{\omega^{4}+(\beta \omega {)}^{2}}$$

Solving the inequality in Equation (5) gives the range for the torsional spring constant k such that

(6) $$0<k<2I\omega^{2}$$

This condition specifies the range of stiffness values for which adding a spring can increase the oscillatory response relative to the no-spring case for a given driving frequency. However, in the present system, the crank-rod mechanism largely constrains the geometric stroke amplitude of the wings. Accordingly, the amplitude in this analysis should be interpreted as a measure of the system’s dynamic compliance, reflected in the torque required to drive the prescribed motion, rather than a direct predictor of stroke magnitude. In addition, because the wing motion is geometrically constrained and non-sinusoidal, the direct application of classical resonance theory, developed for unconstrained, sinusoidally forced oscillators, is limited in this context. Under these constraints, resonance primarily results in reduced torque during stroke reversal, enabling higher flapping frequencies and lower electrical power consumption.

The inequality was therefore introduced with the purpose of qualitatively explaining the conditions under which the addition of a spring can increase the system’s dynamic compliance, rather than serving as a strict design rule. Final spring selection was then determined experimentally by systematically sweeping multiple stiffness values to span the expected resonance frequency range and evaluating thrust, flapping frequency, and efficiency metrics.

## 3. Methods

### 3.1. Experimental Setup—Flapping Test Rig

**Figure 2:**
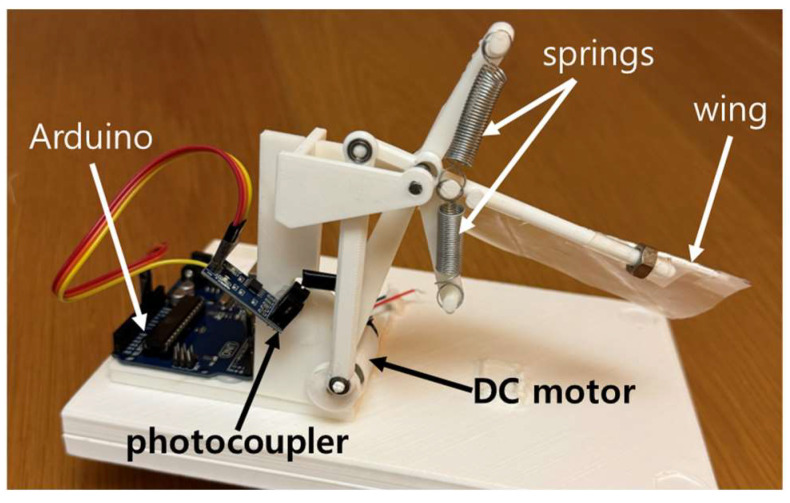
Flapping test rig showing motor, linkage mechanism, wing configuration, and microcontroller-based measurement setup.

To conduct experiments and analyze the flapping motion, a flapping test rig was designed and constructed. The components were modeled in 3D modeling software (Autodesk^®^ Fusion 360, Autodesk, San Francisco, CA, USA [25]) and fabricated using a 3D printer (Single Plus-320C, Cubicon, Seongnam, Republic of Korea [26]), allowing for precise customization of the components. A DC motor, powered by a regulated voltage from a power supply, drives a crank and connecting rod mechanism that produces periodic wing motion. The assembled flapping test rig is shown in Figure 2.

Two rods were mounted above and below the wing hinge to support the attachment of springs. This setup enables the springs to compress and extend in response to the wing’s motion, simulating harmonic oscillation. Four springs with different spring constants were tested: 50, 80, 120, and 160 N/m. These values were selected to span a range expected to shift the system’s natural frequency from below to above the nominal operating flapping frequency, guided by the harmonic-oscillator estimate in Section 2.2 and the practical availability of lightweight springs.

To maintain consistent inertia, a 3 g nut was attached at the wing tip, representing tip mass, for all trials. The wings were constructed using thin plastic membranes to introduce drag, weighing less than 1 g. The wing length was 7.5 cm, with the tip mass located 7 cm from the axis, and the spring positioned 1.6 cm from the axis.

**Figure 3:**
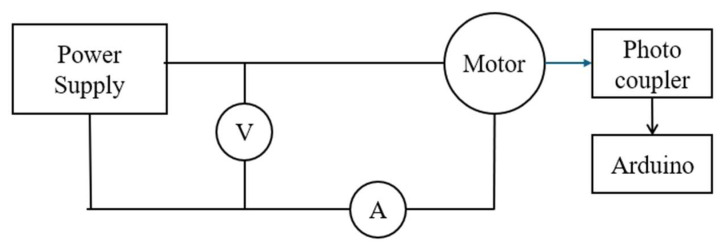
Circuit diagram of flapping test rig.

Power consumption during operation was recorded by measuring voltage and current from the power supply. Additionally, flapping frequency (flaps per second) was measured using a photocoupler by detecting the movement of the crank in the mechanism. The photocoupler was connected to a computer via an Arduino microcontroller, facilitating data acquisition and analysis. The drive electronics and sensing layout are shown in Figure 3.

### 3.2. Thrust Measurement Setup

**Figure 4:**
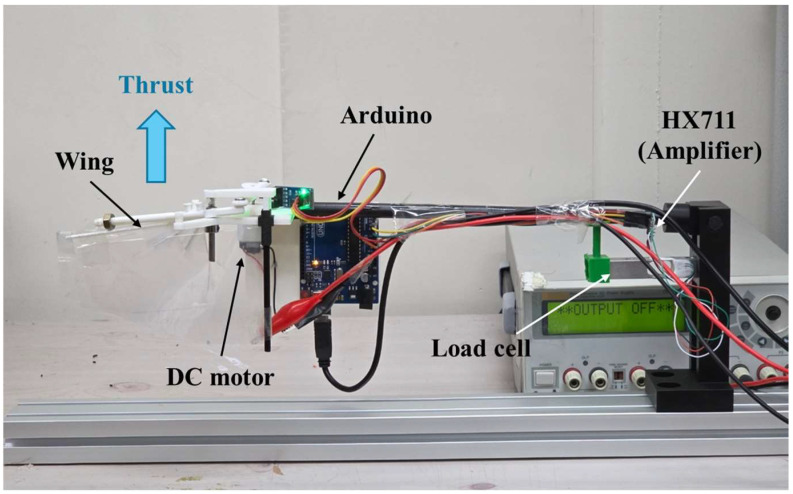
Thrust measurement setup integrated with the flapping test rig.

To directly characterize aerodynamic output, thrust generated by the flapping-wing mechanism was measured using a load cell integrated into the experimental setup. The flapping test rig described in Section 3.1 was mounted at the end of a rigid rod of length 30 cm, and a bar-type load cell was installed 6 cm from the pivot, providing a fivefold amplification of the measured thrust, as shown in Figure 4.

A 1-kg load cell (TAL226, HT Sensor Technology, Xi’an, China), an instrumentation amplifier (HX711, AVIA Semiconductor, Xiamen, China), and an Arduino microcontroller were used for signal acquisition. The load cell has a specified combined error of ±0.02% full scale (FS), corresponding to a static load uncertainty of approximately ±0.2 g at full scale. The specified creep over 30 min is ≤±0.002% FS, and the temperature effect on span is ≤±0.002% FS over a compensated temperature range of −10 to 40 °C. Because all measurements were conducted indoors with short acquisition windows (10 s per condition) and minimal temperature variation, the effects of creep and thermal drift were considered negligible.

All spring configurations were tested using the same load cell, lever amplification ratio, and mounting setup to ensure consistent systematic effects across conditions. Since flapping-induced vibrations may couple into the load-cell signal, thrust data were recorded over a fixed time window and time-averaged to mitigate dynamic effects. For each operating condition, 10 repeated acquisitions were averaged, and this 10-sample mean was repeated three times to obtain a final averaged thrust value. For each power setting, the electrical input power was controlled using the same regulated power-supply approach as in the rig tests described in Section 3.1.

### 3.3. Commercial Ornithopter Platform

A commercially available flapping-wing UAV (MetaFly, Bionic Bird, Marseille, France) was used as a flight-test platform for evaluating the proposed spring-assisted mechanism. The key specifications of the platform are summarized in Table 1.

**Figure 5:**
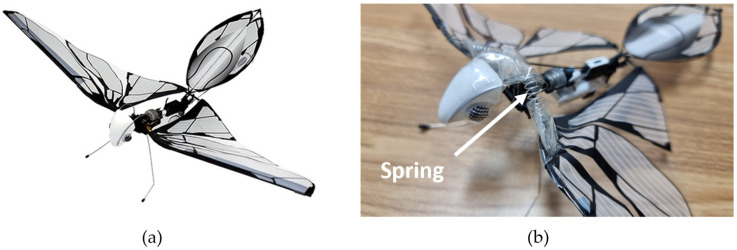
Ornithopter platform configuration comparison: (**a**) Original MetaFly; (**b**) Modified MetaFly with integrated spring mechanism.

To integrate the spring mechanism, a thin wire support was added to the body, extending to the wings, and a spring was then installed between the support structure and the wing mechanism. This modification allows the spring to compress and extend in synchronization with the wing’s motion, effectively storing and releasing elastic energy to improve energy efficiency. The implemented wire support and torsional spring are highlighted in Figure 5.

### 3.4. Wing Trajectory Measurements

The wing trajectory was analyzed to examine differences in flapping behavior with and without the spring mechanism. Theoretically, without a spring, the system acts as a driven, damped rotor without a restoring element, which increases inertial losses at direction reversals.

**Figure 6:**
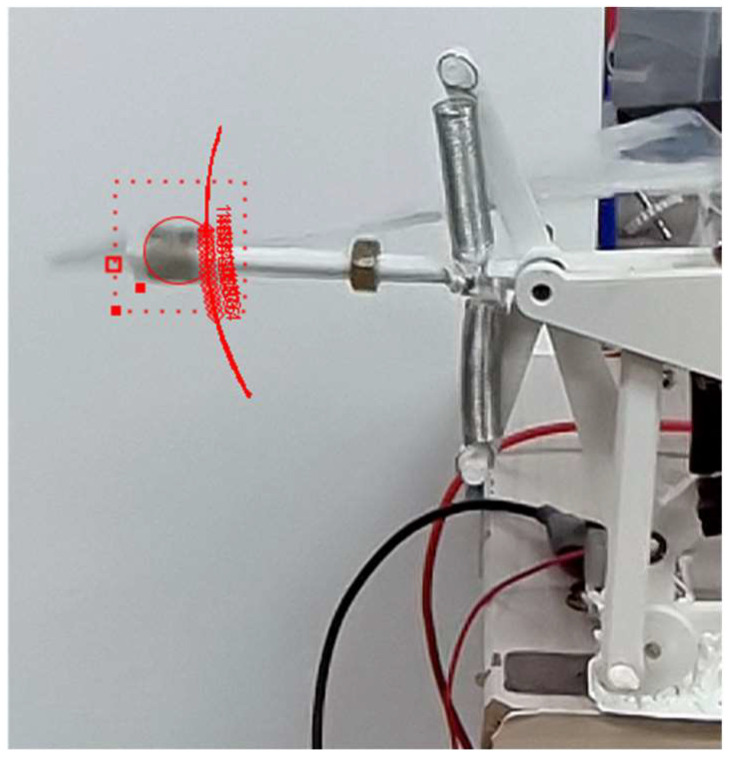
Wing trajectory tracking using a high-speed camera and image processing software Tracker.

The wing motion was recorded using a high-speed camera at 960 fps (frames per second) while the rig was operating. The recorded footage was then processed using the open-source video analysis software, Tracker (version 6.2.0) [27], to track the trajectory of the wings and quantify kinematic parameters, including the flapping frequency f and the peak-to-peak stroke amplitude A. Figure 6 shows the experimental setup and representative image processing with Tracker.

To compare the effects of the spring mechanism, an identical set of experiments was conducted without the spring. The results were analyzed to determine changes in trajectory patterns and energy efficiency. Additionally, the natural frequency of the spring-installed wing system was measured separately through free oscillation tests.

### 3.5. Efficiency Analysis

The energetic performance of the flapping-wing system was evaluated experimentally for configurations with and without an integrated torsional spring and across variations in spring constants. Electrical input power was computed as P=VI where V is the supplied voltage and I is the measured current. For each operating condition, the supplied voltage was increased in discrete steps, and the resulting current, flapping frequency, and thrust were recorded. Flapping frequency was determined as the time-averaged value over a 10-s measurement window. All experiments were repeated three times under identical conditions, and the averaged values were used for analysis.

To evaluate performance, two complementary efficiency metrics were defined. Thrust efficiency, treated as the primary metric of aerodynamic performance, was defined as

(7) $$\eta_{T}=\frac{T}{P}$$

where T is the time-averaged thrust generated by the flapping mechanism. This metric directly quantifies useful aerodynamic force production per unit electrical input power and is therefore most relevant to flight performance and endurance.

In addition, a kinematic efficiency metric was defined as

(8) $$\eta_{K}=\frac{f}{P}$$

where f is the measured flapping frequency. This metric characterizes how effectively electrical input power is converted into sustained wing motion and highlights the mechanical effects of resonance-assisted flapping, particularly the reduction in actuator effort during stroke reversal. While kinematic efficiency does not directly represent aerodynamic performance, it serves as a useful complementary indicator of resonance-induced mechanical benefits.

For each spring configuration, thrust, thrust efficiency, flapping frequency, and kinematic efficiency were analyzed as functions of electrical input power and compared against the no-spring baseline to quantify the performance gains achieved through passive resonance tuning.

### 3.6. Flight Test Procedure

The actual flight test was conducted using the commercial ornithopter to verify whether the proposed spring-assisted design effectively improves flight endurance despite the added weight of the spring mechanism. This evaluation aimed to confirm the practical benefit of the approach in terms of flight efficiency in a realistic operating environment.

To minimize external disturbances such as wind, the test was conducted indoors under controlled conditions. Flight time was measured for both configurations (with and without the spring mechanism), starting from a fully charged battery (4.2 V) until it depleted to the cut-off voltage (3.5 V) at which point the protection circuit automatically shut down the power. Six flights were performed for each configuration to ensure reliability and provide robust comparative data.

## 4. Experimental Results

### 4.1. Wing Trajectory Analysis

**Figure 7:**
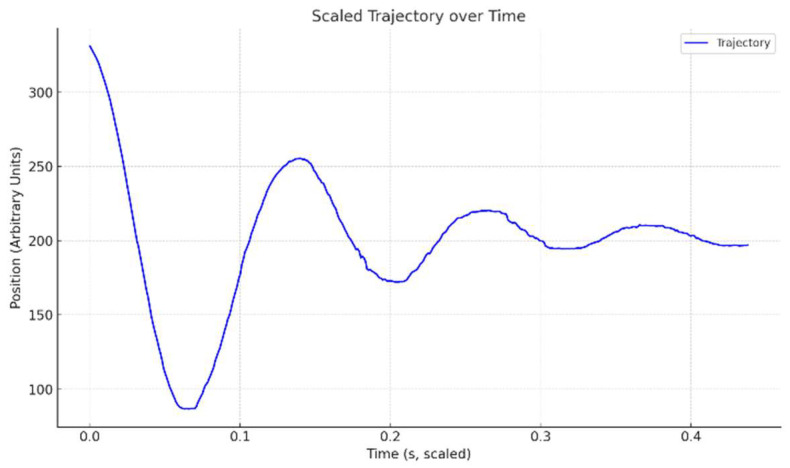
Free response trajectory of the wingtip to measure the natural frequency experimentally.

Figure 7 presents the free response of the wing with the 120 N/m spring attached. From the plot, the period is measured to be approximately 0.14 s, corresponding to a resonant frequency of about 7.1 Hz.

This result is consistent with the theoretical calculation based on [28]

(9) $$f=\frac{1}{2\pi }\sqrt{\frac{k_{\theta }}{I}}=\frac{1}{2\pi }\sqrt{\frac{kl_{s}^{2}}{mL^{2}}}$$

where $k_{\theta }$ denotes the torsional stiffness and I the moment of inertia about the hinge. Conversion of a linear spring to a torsional spring gives $k_{\theta }=kl_{s}^{2}$ when a linear spring of k is located $l_{s}$ from the rotation axis. The moment of inertia of the wing is given by $I=mL^{2}$ when it is modeled as a point mass at a distance L.

Using the parameter values of the flapping test rig described in Section 3.1 (m = 0.003 kg, L = 0.07 m, $l_{s}$ = 0.016 m) and applying Equation (9), the theory-based natural frequency for a spring constant of k = 120 N/m is calculated to be 7.28 Hz, which is in close agreement with the experimentally measured value of 7.1 Hz. As expected, the measured periods and corresponding flapping frequencies varied with spring constant: 0.12 s (8.1 Hz) for 160 N/m, 0.16 s (6.3 Hz) for 80 N/m, and 0.18 s (5.5 Hz) for 50 N/m. These results confirm that the stiffness range selected in Section 3.1 adequately spans the expected resonance region of the system.

**Figure 8:**
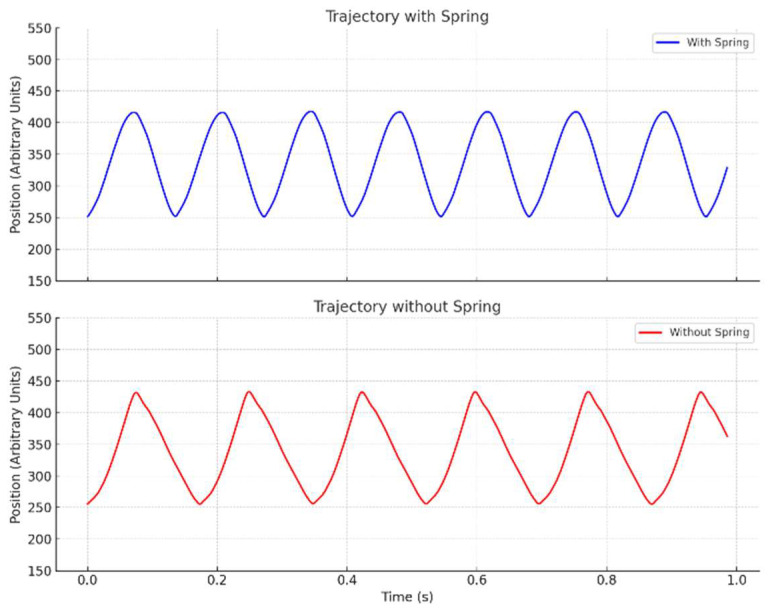
Comparison of wingtip trajectories with the 120 N/m spring and without a spring.

Figure 8 shows the wingtip trajectories with and without the spring (120 N/m). With the spring installed, the trajectory follows a smooth sine wave, whereas the no-spring case exhibits sharp peaks during the reversal of the wing motion, indicating higher inertial loss.

This comparison is performed under identical electrical input rather than identical flapping frequency to illustrate how each mechanism responds to the same actuation effort at the system level. As a result, the spring-assisted case achieves a higher flapping frequency. Although this is not a frequency-matched kinematic comparison, the figure can still be qualitatively interpreted to show the by illustrating the smoother stroke reversal enabled by the spring, consistent with reduced mechanical losses and improved energy efficiency.

### 4.2. Thrust and Thrust Efficiency

**Figure 9:**
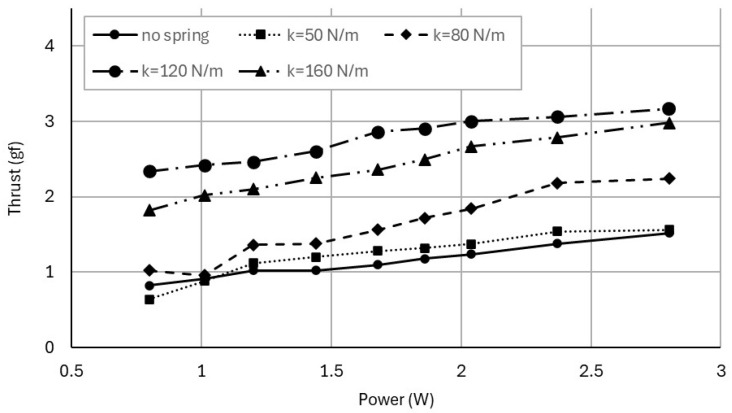
Relationship between electrical input power and measured thrust for the no-spring and spring-assisted configurations, demonstrating enhanced aerodynamic force production with resonance-turned springs.

To evaluate aerodynamic performance, thrust was measured as a function of electrical input power using the lever-amplified load cell setup described in Section 3.2. Figure 9 shows the relationship between power consumption (W) and measured thrust (gram-force; gf) for each configuration. For a given power input, spring-assisted configurations generate higher thrust than the no-spring case, indicating that the resonance tuning method enhances aerodynamic force production. Among the tested configurations, the 120 N/m spring consistently produces the highest thrust across the operating range.

**Figure 10:**
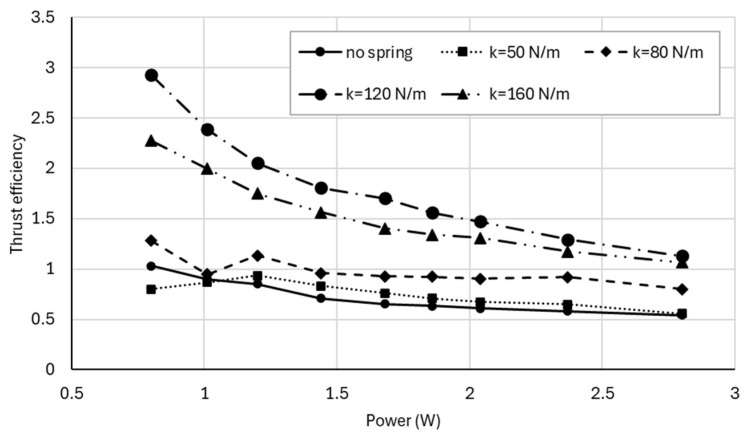
Thrust efficiency (thrust per unit electrical power) as a function of electrical input power for the no-spring and spring-assisted configurations, highlighting improved aerodynamic efficiency for resonance-tuned springs.

To normalize thrust generation, thrust efficiency $\eta_{T}$, defined in Equation (7) as thrust per unit electrical power, is shown in Figure 10. Overall, thrust efficiency decreases with increasing power due to increasing aerodynamic and mechanical losses. However, spring-assisted configurations maintain a clear advantage over the no-spring case throughout the measured range.

**Figure 11:**
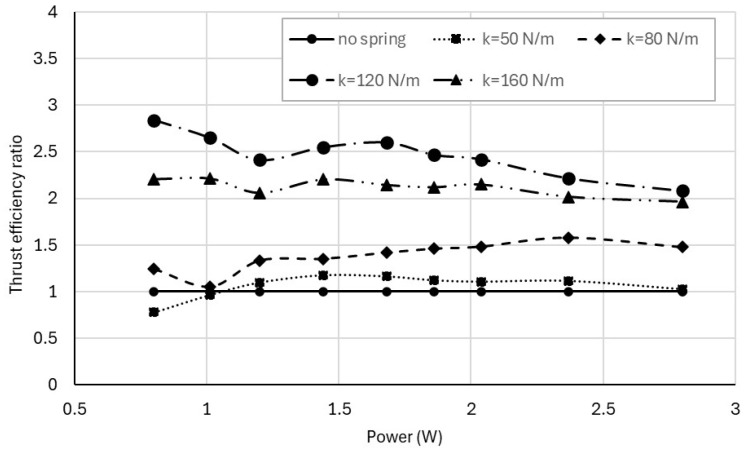
Thrust efficiency ratio (normalized by the no-spring baseline) as a function of electrical input power for the spring-assisted configurations.

The relative improvement in thrust efficiency is quantified in Figure 11, which shows the thrust efficiency ratio normalized by the no-spring baseline. Resonance-tuned configurations provide substantial gains, particularly at lower power levels, with thrust efficiency reaching nearly three times that of the no-spring case for the 120 N/m spring. As power increases, the efficiency ratio gradually decreases, indicating diminishing returns as the system moves away from its optimal resonance condition.

### 4.3. Flapping Frequency and Kinematic Efficiency

**Figure 12:**
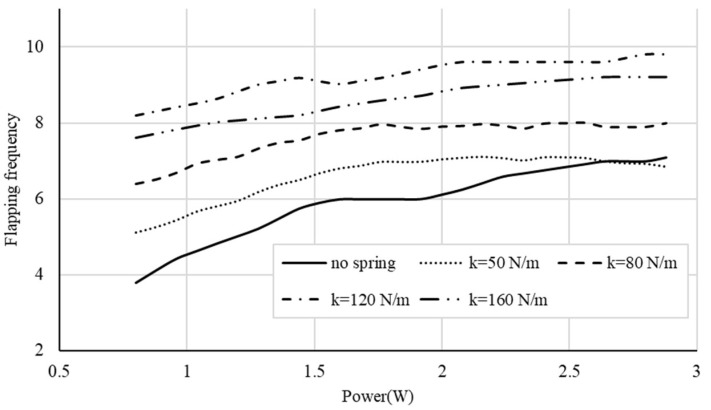
Relationship between electrical input power and flapping frequency for the no-spring and spring-assisted configurations with different stiffness values.

The mechanical response of the flapping system is examined through flapping frequency and kinematic efficiency. Figure 12 shows the relationship between electrical input power (W) and flapping frequency (flaps per second) for each configuration: no spring and springs of 50, 80, 120, and 160 N/m. Spring-assisted systems consistently achieve higher flapping frequencies than the no-spring case. The 120 N/m spring provides the largest increase, particularly at lower power levels, followed by 160 N/m, 80 N/m, and 50 N/m.

As shown in Equation (9), the natural frequency of the rotational system increases with the spring constant. A higher natural frequency shifts the system closer to its resonance condition, which assists stroke reversal by returning stored elastic energy, thereby reducing the required motor torque. Since the speed of a DC motor increases as the torque load decreases, a reduction in this required torque allows the motor to rotate faster, leading to a higher flapping frequency. In contrast, the lowest-stiffness spring (50 N/m) exhibits lower flapping frequencies than the no-spring configuration at higher power levels because it might not store sufficient elastic energy to assist stroke reversal in this regime.

**Figure 13:**
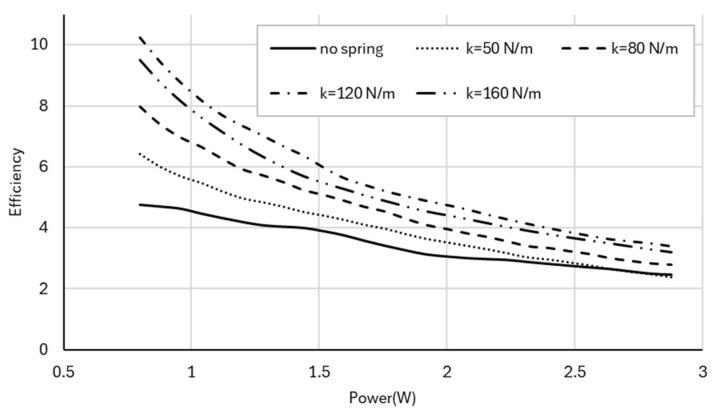
Kinematic efficiency (flapping frequency per unit electrical power) as a function of electrical input power for the no-spring and spring-assisted configurations, illustrating the effect of spring stiffness on frequency-to-power performance.

Kinematic efficiency $\eta_{K}$, defined in Equation (8) as flapping frequency per unit electrical power, is shown in Figure 13. Although kinematic efficiency decreases with increasing power for all configurations, spring-assisted cases consistently outperform the no-spring baseline.

**Figure 14:**
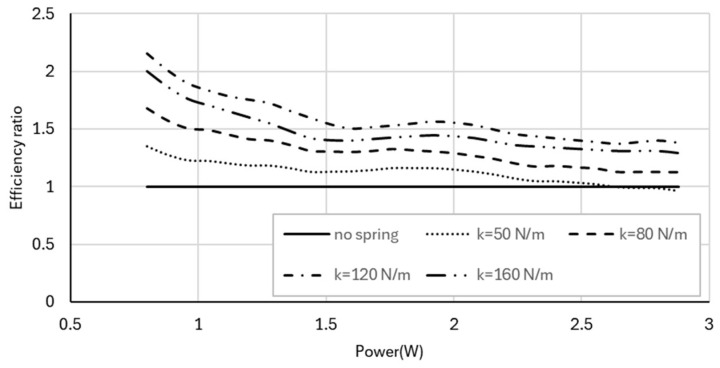
Kinematic efficiency ratio (normalized by the no-spring baseline) as a function of electrical input power for the spring-assisted configurations.

The relative improvement in kinematic efficiency is shown in Figure 14, where the 120 N/m spring again exhibits the largest gains, around a two-fold improvement at lower power levels.

These results confirm that the proposed resonance-tuning method not only amplifies the effective aerodynamic force, as evidenced by the thrust efficiency results, but also enhances kinematic performance.

### 4.4. Flight Test Results

**Figure 15:**
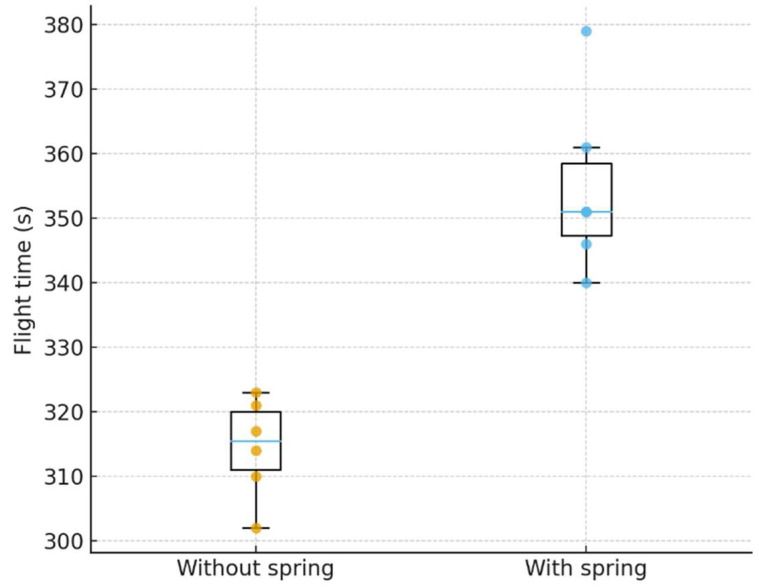
Flight time distribution from actual flight tests of the MetaFly platform without and with the torsional spring (n = 6 per configuration). Two trials with the spring configuration recorded identical times of 351 s.

The distribution of flight times for the two configurations is shown in Figure 15.

The average flight time without a spring was 314.5 s, whereas integrating the spring mechanism extended the flight time to 354.7 s, representing a 12.8% improvement. Although the variation in flight time slightly increased with the spring, all trials showed longer flight durations compared to the no-spring case. To assess statistical significance, a two-sample Welch’s *t*-test assuming unequal variances was performed on the six independent flights for each configuration. The results indicate that the spring-assisted configuration yields a statistically significant increase in mean flight endurance (*p* = 0.00027), confirming that the observed improvement is not attributable to random variation but reflects a consistent enhancement in energy efficiency.

## 5. Discussion

This work demonstrates that a passive spring-assisted mechanism can be used to mechanically tune a flapping-wing system toward resonance, thereby reducing the motor effort required to drive the wings. These results complement computational and control-based energy optimization approaches by showing that passive mechanical tuning alone can shift the system toward a more efficient operating condition. Whereas trajectory optimization reduces power by shaping wing motion or scheduling phases, the proposed approach reduces the required power for a given motion by aligning the structure’s natural frequency with the driving frequency. This finding reinforces prior insights that elastic energy storage can offset energy losses at reversal and demonstrates a practical implementation on a commercially available ornithopter platform.

Among the tested configurations, the 120 N/m spring produced the highest performance but should be interpreted as the condition closest to resonance within the tested stiffness range (50, 80, 120, and 160 N/m), rather than as a global optimum. Identifying an exact optimal stiffness value was not the primary objective of this work; instead, the focus was on demonstrating how resonance-tuned passive compliance influences system-level efficiency trends.

Lift was not directly measured in this study. Thrust was used instead as a relative, system-level aerodynamic performance metric because the experiments compared spring stiffness under identical wing planform, mass distribution, and kinematic constraints imposed by the crank–rod mechanism. Under these controlled conditions, both thrust and lift tend to scale with flapping frequency and the temporal characteristics of wing motion, such that increases in thrust-to-power can be qualitatively associated with improved lift generation. The observed improvements in thrust-to-power efficiency were accompanied by increased flight endurance, indicating that lift performance was not adversely affected. Therefore, thrust still provides a meaningful relative metric for assessing system-level performance in the present context, while the lack of direct lift measurement remains a limitation.

In the commercial ornithopter flight tests, the integration of the spring and wire support introduced an additional mass of approximately 0.3 g (0.15 g per wing), corresponding to about 3.1% of the total airframe mass (9.5 g). The wire support was designed with minimal cross-sectional area to limit additional aerodynamic drag. Despite this mass penalty, the spring-assisted configuration achieved a statistically significant 12.8% increase in flight endurance, indicating that the resonance-induced reduction in mechanical energy consumption outweighs the added mass at the system level. While the individual contributions of added mass, drag increase, and mechanical energy savings were not independently separated, the observed endurance gain reflects a net efficiency benefit under the tested conditions. This result suggests that low-complexity passive resonance tuning may provide a practical pathway to improving endurance in small, battery-constrained flapping-wing UAVs.

Several additional limitations of this study should be noted. First, endurance validation was performed on a single commercial airframe in an indoor environment; results may vary with different airframe configurations or under outdoor conditions, so the reported gain should be interpreted as specific to this platform. Second, while thrust measurements were included to characterize aerodynamic force production and efficiency, no direct measurements of lift or aerodynamic torque were conducted, limiting a more complete separation of inertial, mechanical, and aerodynamic loss contributions. Third, resonance characterization focused on one operating point; payload changes, wing compliance, or long-term structural changes were not examined.

Future work will include (i) incorporating torque and lift measurements to further separate mechanical and aerodynamic contributions, enabling a more detailed interpretation of the observed changes in power consumption and aerodynamic performance; (ii) implementing closed-loop resonance tracking to account for battery voltage sag and structural drift; and (iii) investigating scalability to smaller vehicles and flexible wings, including adjustable or modular spring mechanisms for rapid field tuning.

## 6. Conclusions

In this paper, we investigated the integration of a passive spring mechanism into flapping-wing ornithopters to enhance energy efficiency and aerodynamic performance. Modeling the wing as a harmonic oscillator showed that properly tuned spring stiffness can induce resonance, resulting in smoother wing motion and reduced energy loss. Experimental results demonstrated that spring-assisted configurations produced nearly threefold higher thrust efficiency and achieved up to a twofold improvement in kinematic efficiency relative to the no-spring baseline, indicating enhanced aerodynamic output per unit electrical power. Flight tests on a commercial ornithopter further confirmed the practical benefit of this approach, yielding a 12.8% increase in average flight endurance. Direct thrust measurements further verified that the efficiency gains were accompanied by increased aerodynamic force production rather than purely kinematic effects. These results highlight the potential for passive, spring-assisted designs to overcome key limitations of conventional ornithopters and provide a foundation for more efficient bio-inspired UAVs.

## Figures and Tables

**Table 1:** MetaFly platform specifications.

Parameter	Value
Length	0.19 m
Wingspan	0.29 m
Weight	<0.01 kg
Battery	Li-Po, 0.058 Ah
Flight time	up to 8 min
Maximum speed	5 m/s

## Data Availability

Data is contained within the article.

## Abbreviations

The following abbreviations are used in this manuscript:

UAV	Unmanned Aerial Vehicle
FWMAV	Flapping-Wing Micro Air Vehicle
